# Iodine Deficiency-Induced Thyrotoxicosis Mimicking Graves’ Disease: A Case of Triiodothyronine (T3)-Predominant Hyperthyroidism Without Goiter

**DOI:** 10.7759/cureus.95370

**Published:** 2025-10-25

**Authors:** Pooja Alipuria, Atush Alipuria

**Affiliations:** 1 Internal Medicine, Yashoda Super Speciality Hospital, Kaushambi, Ghaziabad, IND; 2 Pulmonology and Critical Care, Yashoda Super Speciality Hospital, Kaushambi, Ghaziabad, IND

**Keywords:** graves' disease, hyperthyroidism, iodine deficiency, non-iodized salt, t3, thyroid scintigraphy, thyrotoxicosis, triiodothyronine

## Abstract

Triiodothyronine (T3)-predominant thyrotoxicosis without goiter is uncommon, with differential diagnoses including Graves’ disease, thyroiditis, and iodine deficiency-related autonomy. Diagnostic certainty can be challenging in resource-constrained settings. We describe a 41-year-old man who presented with palpitations, heat intolerance, and weight loss and reported exclusive use of non-iodized rock salt for three years. Examination revealed tachycardia and tremor without goiter or orbitopathy. Thyroid function tests showed suppressed thyroid-stimulating hormone, elevated free T3, normal free thyroxine, and negative thyroid autoantibodies. Ultrasound demonstrated a diffusely enlarged, non-nodular gland with normal echogenicity, while scintigraphy revealed diffuse homogeneous uptake. He was treated with carbimazole (20 mg/day, tapered to 5 mg) and propranolol (40 mg/day, later withdrawn) and was advised to switch to iodized salt at an intake of approximately 5 g/day. After nine months, the patient discontinued carbimazole on his own; two months later, thyroid function was normal, and he remained euthyroid over 24 months of follow-up without medication. Although thyrotropin receptor antibody-negative Graves’ disease was initially suspected, the combination of sustained remission after therapy cessation, negative antibodies, and exclusive rock salt use supported a diagnosis of iodine deficiency-induced thyrotoxicosis. This case highlights the importance of dietary history and structured follow-up in evaluating atypical thyrotoxicosis and illustrates how iodine deficiency can mimic autoimmune hyperthyroidism in clinical practice.

## Introduction

Iodine deficiency remains one of the most widespread nutritional disorders globally, affecting more than two billion people and contributing substantially to thyroid dysfunction [1]. In iodine-deficient states, thyroid hormone synthesis shifts toward triiodothyronine (T3), which requires fewer iodine atoms than thyroxine (T4) [2]. This physiological adaptation explains why chronic iodine deficiency may predispose individuals to T3-predominant thyrotoxicosis [2]. Prolonged deficiency can also lead to thyroid autonomy and toxic nodular goiter, particularly in older adults [3,4]. Even minor regional differences in iodine intake can significantly influence the prevalence and patterns of thyroid disorders [5].

Despite the implementation of universal salt iodization programs, mild iodine deficiency persists in some parts of India, especially in the Himalayan foothill states and regions of North and Northwest India, such as Uttarakhand, Himachal Pradesh, and Rajasthan, where non-iodized rock salt remains commonly used due to cultural and taste preferences [6]. In contrast, most coastal regions demonstrate near-adequate iodine sufficiency. In iodine-deficient areas, thyroid hyperfunction resulting from diffuse or nodular autonomy may present with biochemical and scintigraphic features that closely mimic autoimmune thyrotoxicosis, complicating diagnosis [7,8]. Graves’ disease, the most common cause of thyrotoxicosis worldwide, is characterized by autoimmune stimulation of thyrotropin (thyroid-stimulating hormone, TSH) receptors and is typically managed with antithyroid medications, radioiodine therapy, or surgery [9].

We report a case of T3-predominant thyrotoxicosis without goiter in a middle-aged man who had exclusively used non-iodized rock salt for several years. Sustained remission following iodine repletion and early discontinuation of antithyroid therapy supports iodine deficiency-induced thyrotoxicosis as the most likely etiology.

## Case presentation

A 41-year-old vegetarian male with a sedentary occupation from North India presented with a six-month history of palpitations, heat intolerance, irritability, and unintentional weight loss of approximately 5 kg. His symptoms developed gradually and progressed over four months before medical evaluation. He had no history of medical or psychiatric illness, no family history of thyroid disease, and was not taking any regular medications or dietary supplements. He denied the use of herbal preparations or exposure to pesticides, industrial chemicals, or other substances known to influence thyroid function. Notably, he reported using only non-iodized rock salt for cooking and seasoning for the past three years.

On examination, his pulse rate was 96 beats per minute, and fine tremors were noted, but there was no evidence of goiter or orbitopathy. Cardiovascular, respiratory, and abdominal examinations were unremarkable. Thyroid function tests revealed suppressed TSH, elevated free T3, and normal free T4. Both the thyrotropin receptor antibody (TRAb) and anti-thyroid peroxidase antibody tests were negative. Other biochemical parameters, including liver and renal function, were within normal limits (Table 1).

**Table 1:** Laboratory investigations with reference ranges. ALT, alanine aminotransferase; AST, aspartate aminotransferase; ESR, erythrocyte sedimentation rate; FT3, free triiodothyronine; FT4, free thyroxine; TLC, total leukocyte count; TRAb, thyrotropin receptor antibody; TPO, thyroid peroxidase; TSH, thyroid-stimulating hormone

Parameter	On presentation	Repeated (to confirm)	On follow-up (after six weeks)	On follow-up (after three months)	On follow-up (after six months)	On follow-up (after one year)	On follow-up (after two years)
TSH (0.3-5.6 mIU/L)	<0.004	0.004	0.01	7.83	2.28	1.2	1.25
FT3 (0.9-3.6 pg/mL)	3.81	3.81	2.56	0.855	3.46	3.3	-
FT4 (0.6-1.8 ng/dL)	1.12	1.11	0.9	0.54	1.05	1.08	1.15
TRAb (negative: <1.00 IU/L; equivocal: 1.10-1.50 IU/L; positive: >1.5 IU/L)	-	0.91	-	-	-	-	-
Anti-TPO antibodies (<5.61 IU/mL)	-	3.41	-	-	-	-	-
ESR (0-20 mm/hr)	-	5	-	-	-	-	-
Hemoglobin (13.0-17.0 g/dL)	-	14.2	-	14.6	-	-	14.8
TLC (4000-10,000 /cumm)	-	5250	-	5540	-	-	4590
Platelet (1.50-4.10 lakh/cumm)	-	2.37	-	3.7	-	-	3.5
Urea (16.60-48.50 mg/dL)	-	23	-	25	-	-	22
Creatinine (0.70-1.20 mg/dL)	-	0.7	-	0.84	-	-	0.72
Total bilirubin (0.2-1.2 mg/dL)	-	0.3	-	0.5	-	-	0.4
Direct bilirubin (<0.3 mg/dL)	-	0.2	-	0.2	-	-	0.2
AST (5-34 U/L)	-	23	--	25	-	-	23
ALT (0-41 U/L)	-	19	-	18	-	-	17
Fasting blood sugar (<100 mg/dL)	-	92	-	-	-	-	94

**Figure 1:**
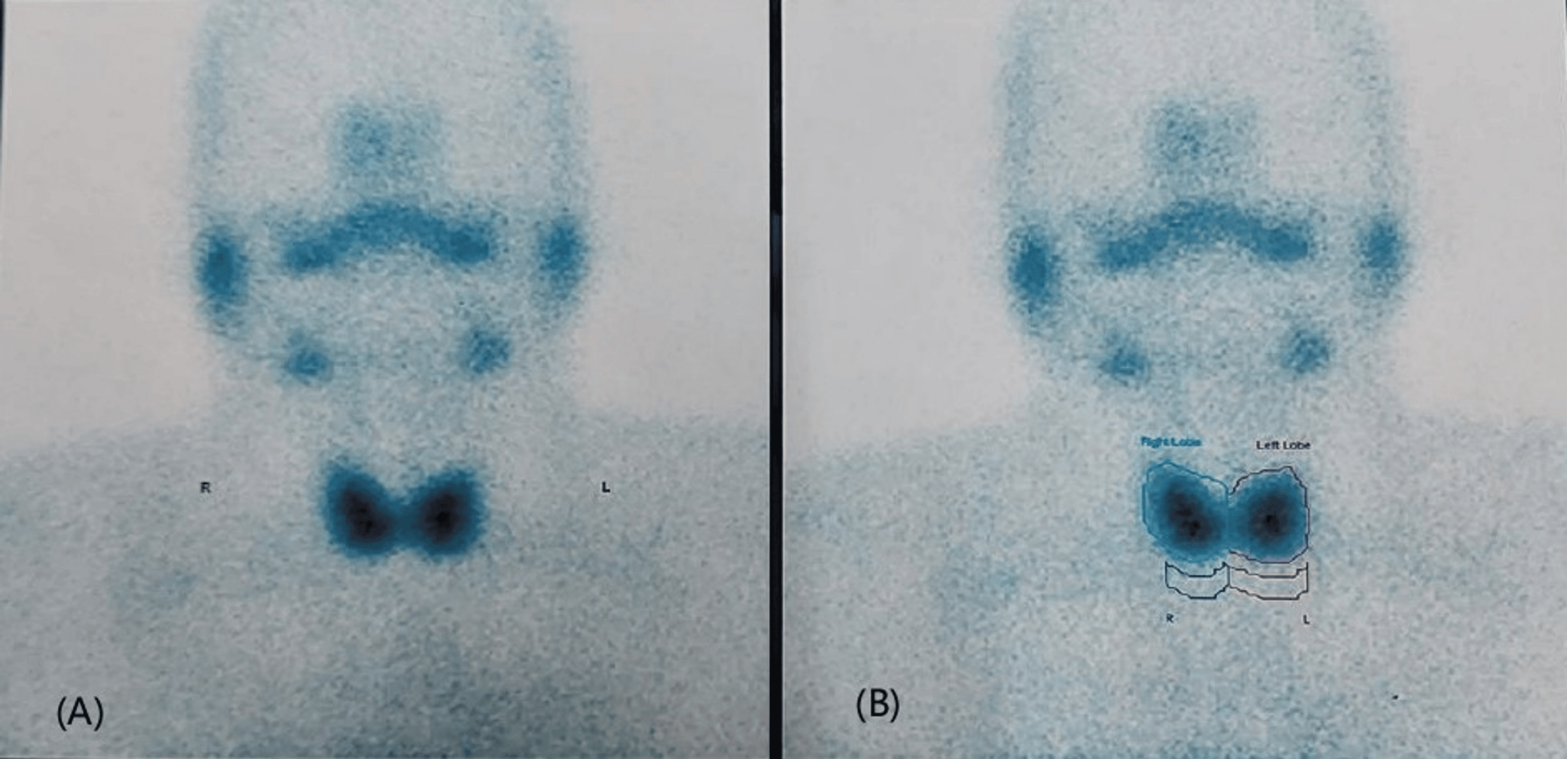
Thyroid scintigraphy with technetium-99m pertechnetate. (A) Anterior planar image showing diffuse, homogeneous tracer uptake in both lobes of the thyroid gland. (B) Same image with regions of interest drawn around each lobe for uptake quantification, showing a calculated thyroid uptake of 1.2%.

Ultrasound of the thyroid demonstrated a diffusely enlarged, homogeneous gland with normal echogenicity and no nodules. Thyroid scintigraphy using technetium-99m pertechnetate revealed diffuse, homogeneous tracer uptake of 1.2%, which remained preserved despite suppressed TSH (Figure 1).

These findings initially suggested TRAb-negative Graves’ disease. The patient was started on carbimazole 20 mg/day and propranolol 40 mg/day (in two divided doses), along with dietary iodine repletion by replacing rock salt with iodized salt (~5 g/day). Carbimazole was tapered to 10 mg at six weeks and to 5 mg at three months based on thyroid function test results. Propranolol was gradually tapered and discontinued once the resting heart rate stabilized below 90 beats per minute and symptoms resolved, in accordance with the 2016 American Thyroid Association guidelines for the management of hyperthyroidism [9].

Although the patient’s clinic visits were less frequent than advised, he was compliant with carbimazole and regularly underwent thyroid function testing at each visit. At each review, he was specifically asked about medication adherence and consistently reported taking carbimazole as prescribed for nine months, after which he discontinued it on his own. Two months later, repeat thyroid function tests were normal. He remained clinically and biochemically euthyroid during 24 months of follow-up without medication, maintaining only dietary iodized salt. A summary of laboratory values and the clinical timeline is provided in Table 1 and Table 2.

**Table 2:** Clinical timeline of presentation, treatment, and follow-up. T3, triiodothyronine; TFT, thyroid function test; TRAb, thyrotropin receptor antibody; TPO, thyroid peroxidase

Time (months)	Key events
-6 to 0	Gradual onset of palpitations, heat intolerance, irritability, and ~5 kg weight loss.
0	Diagnosed with T3-predominant thyrotoxicosis (TRAb and anti-TPO negative). Started carbimazole 20 mg/day and propranolol 40 mg/day and advised to switch to iodized salt (~5 g/day).
1.5 to 3	Carbimazole tapered to 10 mg/day and then to 5 mg/day based on TFTs. Propranolol tapered with heart rate monitoring and discontinued once resting HR <90 bpm and symptoms resolved.
3 to 9	Maintained carbimazole 5 mg/day with good adherence.
9	Patient self-discontinued carbimazole after nine months of therapy.
11	Thyroid function normalized; patient euthyroid.
11 to 33	Sustained euthyroidism for 24 months on iodized salt alone.

## Discussion

The differential diagnosis of T3-predominant thyrotoxicosis without goiter includes Graves’ disease, thyroiditis, and iodine deficiency-related autonomy [2-4]. Graves’ disease typically presents with diffuse homogeneous uptake on scintigraphy, diffuse non-nodular thyroid enlargement, and hypoechogenicity with hypervascularity on ultrasound [2]. In this patient, the absence of orbitopathy, negative thyroid antibodies, and sustained remission despite early discontinuation of carbimazole argued against Graves’ disease. In suppressed-TSH states, a pertechnetate uptake of approximately 1-2% is considered indicative of thyroid autonomy, as observed in this patient (1.2%), whereas Graves’ disease usually demonstrates uptake values exceeding 2% [7,9].

Iodine deficiency-related autonomy remained the most plausible etiology. The patient’s exclusive use of non-iodized rock salt provided a clear dietary basis for iodine deficiency, consistent with reports of persistent mild iodine deficiency in parts of India despite national iodization programs [6]. In deficiency states, a T3-predominant profile is expected because T3 synthesis requires fewer iodine atoms [2]. The preserved homogeneous uptake (1.2%) with suppressed TSH fits the pattern of diffuse or disseminated autonomy, in which the entire gland exhibits mild hyperfunction without nodularity [7,8]. Chronic iodine deficiency promotes clonal thyrocyte expansion, leading initially to diffuse autonomy that may later evolve into nodularity [10]. Sustained euthyroidism following iodine repletion and withdrawal of antithyroid therapy further supports iodine deficiency-induced thyrotoxicosis rather than Graves’ disease.

Thyroiditis was unlikely, as it typically produces T4-predominant thyrotoxicosis with low or absent uptake on scintigraphy [8,9]. In this case, preserved uptake excluded thyroiditis. Occupational or environmental exposure to thyroid-disrupting pesticides such as organochlorines, organophosphates, and pyrethroids was also ruled out based on history. These compounds have been shown to interfere with thyroid hormone synthesis, metabolism, and receptor signaling [11].

This case highlights the importance of obtaining a detailed dietary history in patients presenting with atypical thyrotoxicosis, particularly regarding iodized salt use in regions where mild iodine deficiency persists. It illustrates how iodine deficiency can mimic autoimmune hyperthyroidism, presenting with T3-predominant thyrotoxicosis, diffuse homogeneous uptake, and negative antibodies. Regular monitoring and structured follow-up are essential to confirm remission and prevent overtreatment, while awareness of iodine intake variability can help avoid unnecessary prolonged antithyroid or radioiodine therapy.

The main limitation of this report is the absence of urinary iodine concentration measurement, which would have objectively confirmed iodine deficiency. Ultrasound images were not archived, and repeat scintigraphy to calculate TSH-normalized uptake was not performed. Nevertheless, integration of dietary history, biochemical profile, imaging findings, and long-term follow-up provides a coherent and plausible diagnosis of iodine deficiency-induced thyrotoxicosis.

Table 3 summarizes the distinguishing clinical, imaging, biochemical, and hormonal features of Graves’ disease, iodine deficiency-related autonomy, and thyroiditis.

**Table 3:** Diagnostic approach to T3-predominant thyrotoxicosis without goiter. Key differentiating features of Graves’ disease, iodine deficiency-related autonomy, and thyroiditis, summarized from standard endocrine references [2-4,7,9]. T3, triiodothyronine; T4, thyroxine

Feature	Graves’ disease	Iodine deficiency autonomy	Thyroiditis
Orbitopathy	Often present	Absent	Absent
Autoantibodies	Frequently positive	Negative	Negative
Ultrasound	Diffuse enlargement with increased vascularity and hypoechogenicity	Diffuse enlargement, nodular or non-nodular, normal echogenicity	Diffusely hypoechoic with reduced vascularity
Scintigraphy	High diffuse uptake	Preserved diffuse uptake	Absent uptake
Urinary iodine	Normal	Low	Normal
Hormone pattern	T3- or mixed-predominant	T3-predominant	T4-predominant (higher T4/T3 ratio)
Course with antithyroid drugs	Relapse likely if stopped before 12-18 months	May remit with iodine repletion ± short course of antithyroid drugs	Self-limiting
Long-term outcome	Often requires radioiodine or surgery	May remain euthyroid	Spontaneous resolution

## Conclusions

This case illustrates how iodine deficiency can mimic autoimmune hyperthyroidism, presenting as T3-predominant thyrotoxicosis with diffuse homogeneous uptake despite negative antibodies. Sustained remission following dietary iodine repletion underscores the diagnostic value of follow-up, as such a course is uncommon in Graves’ disease. Although universal salt iodization has reduced iodine deficiency globally, pockets of risk persist due to dietary practices and cultural factors. Clinicians should therefore consider iodine deficiency in the differential diagnosis of atypical thyrotoxicosis and obtain a thorough dietary history before initiating long-term antithyroid therapy.
